# National recruitment scheme for pre-registration pharmacist training in England and Wales: a mixed method evaluation of experiences of applicant pharmacy students

**DOI:** 10.1186/s12909-019-1883-5

**Published:** 2019-12-04

**Authors:** Laura McEwen-Smith, Malcolm James Price, Gail Fleming, Tim Swanwick, Christine Hirsch, Asma Yahyouche, Jonathan Ward, Sharon Buckley, Atif Shamim, Vibhu Paudyal

**Affiliations:** 1 School of Pharmacy, University of Birmingham, B15 2TT, Birmingham, UK; 2 NHS Health Education England, London, UK; 3 Institute of Applied Health Research (Biostatistics), University of Birmingham, Birmingham, UK; 4 Royal Pharmaceutical Society, London, UK

**Keywords:** Pre-registration training, Early career training, Pharmacists, Healthcare training

## Abstract

**Abstract:**

**Background:**

A national pre-registration pharmacist training recruitment scheme, which replaces local recruitment models, was introduced in England and Wales in 2017. The national recruitment system allows pharmacy students to apply for the 52 weeks training programmes (mandatory requirement for registration as a pharmacist), through a single application system prior to undertaking a nationally administered assessment. This study aimed to explore experiences of pharmacy students on the national recruitment scheme, particularly their views on the selection methodology, application process, and offer outcomes.

**Methods:**

This mixed method study involved a) an online survey of all (approximate *n* = 2800) year 4 (final year of MPharm degree) pharmacy students in England and Wales and b) a qualitative focus group with four students. The study population was eligible to participate in the 2017/18 national recruitment scheme. Survey respondents were invited to participate in a focus group. Quantitative data were analysed using descriptive and inferential analysis. Qualitative data were analysed using the framework technique. Participation was voluntary. Ethical approval from University of Birmingham was obtained.

**Results:**

A total of 307 completed surveys were returned (approximate response rate 11%). Respondents were generally satisfied with the application process and commended the fairness of the selection methodology and convenience in allowing them to apply to multiple training providers. Most survey respondents (*n* = 181, 72.9%) were either satisfied or highly satisfied with the training programme they were offered based on their assessment performances. Three themes and eight sub-themes obtained from the analysis of over 200 open comments data from the survey and transcript of a focus group with four participants. Results suggested the need to widen the timeframe available for applicants to shortlist their preferred employers, improve the method of programme listing in the application system, and consideration of prior achievements including academic performances and placement experiences to be included in the selection methodology.

**Conclusions:**

Experiences of pharmacy students on the national recruitment scheme suggest that respondents considered the selection methodology to be fair. Student engagement and satisfaction with the recruitment system can be maximised through improved listing of employers and widening the timescales for students to shortlist their preferred employers during application process. Inclusion of University achievements in the selection methodology will require consideration of evidence based approaches. Low response rate limits generalisation of findings.

## Background

In the UK, the General Pharmaceutical Council (GPhC) of Great Britain requires a person to undertake four years of educational training (normally through Master of Pharmacy Course from an accredited University in the UK), followed by successful completion of a 52 week programme of preregistration training in Great Britain, and pass a registration assessment conducted by the GPhC [1]. The pre-registration training is facilitated by Health Education England’s (HEE) which is a non-departmental public body and aims to support the delivery of healthcare and health improvement to the patients and public of England in ensuring that the workforce has the ‘right numbers, skills, values and behaviours, at the right time and in the right place’ [2]. HEE introduced a Pharmacy Education Reform Programme to improve the quality of pre-registration pharmacist training, a national Pre-registration Pharmacist Training Recruitment Scheme England and Wales in 2017 [3]. The scheme is mandated for all HEE funded posts and optional for community pharmacy places funded by NHS England. In year one, most (2161 of approximately 2800) of pre-registration pharmacist training posts were advertised via this route. In order to recruit via the National Recruitment scheme, all employers agree to abide by the HEE Quality Framework (or Welsh equivalent for employers in Wales) for pre-registration training which includes six quality domains including learning environment and culture, supporting learners and educators and delivering curricula and assessments [4].

Similar to those used for medicine, dentistry and healthcare science, the centralised system of recruitment uses an electronic platform and replaces the previous localised and employer-led recruitment process. A single application for as many available pre-registration training places across the secondary care (i.e. hospital) and community sectors as desired can be submitted. Students can rank their choices, or opt for no preference. Students can also deselect employers or programmes that they do not want to accept.

As part of the selection process, students undertake situational judgement tests (SJT); multiple mini interviews (MMI) including a scenario based exercise and past behavioural interview questions; and pharmaceutical calculations (Table 1). The SJT and MMIs assess candidates against a professional attribute framework [5] including communication and consultation skills, problem solving, clinical analysis, decision-making and professional integrity and ethics. National assessment centres were distributed across various geographical regions of the country. Students are offered a place based on their test performances matched with their shortlisting of pre-registration training programmes and employers. Students can accept an offer (with or without upgrade option if a programme higher in the preference becomes available) or decline an offer.

**Table 1:** Assessment methodology for national pre-registration pharmacist selection process

Multiple mini interviews	Situational Judgement tests	Pharmaceutical Calculations
Includes scenario based questions, reflection on past experiences and problem solving. A total of six exercises to be completed in 40 min with each measuring a different professional attribute.	Assesses applicant judgement in work relevant situations. A total of 52 scenarios to be completed in 104 min measuring four different professional attributes. Students expected to indicate what they should do in response to the situation presented by selecting from the multiple response options given.	Includes assessment of basic pharmaceutical numeracy skills. Students given 10 questions to attempt in 15 min.

Adapted from [2].

Gathering early experiences of the national recruitment scheme will inform future recruitment cycles thereby improving applicant, employer and stakeholder satisfaction and engagement with the process. Apprehension about the competitive nature of the recruitment, lack of information and support may often dissuade students from engaging with the application process. Prior opportunities to interact with training providers can minimise these barriers [6]. International literature shows that higher number of programmes selected by the applicants, female gender and better performances in pharmacy schools positively influence offer outcomes in the recruitment process [7].

There is a lack of literature around student perception of national, competitive selection methodology for early career training around selection methodology, perceived fairness, application process and selection outcomes. A recent international consensus statement on future research and practice priorities on the high-stakes selection methodology identified that future research should explore ‘fairness and accountability for all candidates’ [8]. Limited literature from other clinical disciplines suggest that selection methodology involving SJT and MMIs are often generally regarded by stakeholders including applicants and assessors as ‘fair’ compared to traditional interviews, references and personal statements [9]. However, perception often varies across the stakeholder groups and the data are mostly derived from medical schools admissions, foundations training and residency selection processes [9].

In the context of the newly adopted selection and recruitment methodology for pharmacist pre-registration trainees, this study aimed to explore pharmacy students’ perspectives on the national pre-registration pharmacist training recruitment scheme in England and Wales including their views on the application process, selection methodology and offer outcomes.

## Methods

Data for this study were collected as part of the larger evaluation of the new national pre-registration pharmacist training recruitment scheme. A mixed-method approach was used. These included: Phase 1: a web-based survey of all students undertaking Master of Pharmacy Year 4/Overseas Pharmacist Assessment Programme (OSPAP) who were eligible to apply to the 2017/18 scheme, followed by focus groups with students.

### Phase 1: survey

A whole population sampling (i.e. applicants to the 2017/18 recruitment cycle) method was used. The survey questionnaire consisted of a mix of closed (including the use of Likert-type agree/disagree statements) and open-ended questions. The survey and the topic guide for the qualitative study were designed using existing literature, research team input and discussion amongst the national evaluation steering group members which consisted of student, employer, academic, assessor and HEE representatives. The survey consisted of three sections and 36 questions. All Schools of Pharmacy in England and Wales (addressed to heads of Schools and School pre-registration training recruitment leads) were requested to circulate a link to an online questionnaire. Two reminder emails were sent at two and four week intervals to maximise response rates [10]. Data were analysed using descriptive and inferential statistics using STATA version 15 (College Station, Texas, USA). Statistical comparisons used the chi-square test or chi-square test of trend. Comparisons were made across gender, age and ethnicity demographic variables. A pilot study was not undertaken due to time constraints as the study was was intended to inform the planning for subsequent recruitment cycles.

### Phase 2 methodology

Survey respondents were also asked to express interest in participation in focus groups by providing their contact details in a separate section. The identifiable information was then separated from the rest of the questionnaire responses from the downloaded data. Two focus groups involving 6–8 participants in each group were intended. Where more than the required numbers would express interest, we intended to use maximum variation sampling using gender, ethnicity, preferred employer type and geographical locations. Focus groups were held online by LMS, utilising WebEx. Informed consent was obtained prior to the focus groups. Qualitative data from focus groups and responses from the open-ended questions from the questionnaire were analysed together using the framework technique [11]. A thematic coding framework was developed based on the research aims and objectives and topic guide following familiarisation with the data. Any new emergent themes were added during the analysis. Duplicate independent coding and analysis (LMS and VP) of the qualitative data was undertaken. Field notes were undertaken during focus group. Qualitative data are reported using COREQ guidelines (Additional file 1) [12].

Ethical approval for the study was obtained from University of Birmingham research ethics committee (ERN_17–1399).

## Results

Three hundred and seven completed surveys were received from a total population of approximately 2800 students (approximate response rate: 11%). Mean (SD) age was 22.6 (2.3). Majority of the survey respondents were female (*n* = 234, 76.0%) and of White British or Irish (*n* = 130, 42.3%) followed by Indian (*n* = 44, 14.3%) ethnicity. Of these 295 (96.1%) were applicants to the HEE pre-registration pharmacist training recruitment scheme with the remaining 12 (3.9%) being non-applicants of the scheme who were eligible to complete only some of the questions in the questionnaire. Over 200 respondents provided open-ended comments in the questionnaire when asked for their views on the national recruitment scheme. Although 11 participants had confirmed participation across two sessions, only one focus group was conducted lasting approximately 60 min. A total of four participants took part with the rest withdrawing or not showing up. Further recruitment effort was not deemed necessary as the preliminary analysis of the responses to open ended comments of survey and focus group data provided assumption that data saturation was achieved.

### Selection of pre-registration training providers/programmes

Most respondents (66.2%) reported shortlisting between 1 and 100 programmes with a further 22.7% shortlisting between 101 and 300 programmes (Table 2).

**Table 2:** Number of training prospective programmes applied by the applicants

Number of programmes	n (%)
1–100	198 (66.2%)
101–300	68 (22.7%)
301–500	12 (4%)
501–700	5 (1.7%)
701–900	4 (1.3%)
901–1100	5 (1.7%)
1101–1300	5 (1.7%)
No specific preference	2 (0.7%)
Sectors	
NHS Acute Hospital	284 (37.4%)
Community Pharmacy – Large Chain Multiple	178 (23.4%)
Community Pharmacy – Medium/Small Independent Multiple	105 (13.8%)
Community Pharmacy – Independent	81 (10.7%)
Cross-sector programme (both NHS and Community Pharmacy)	112 (14.7%)
Sectors in top 10 selection by individual applicant	
NHS Acute Hospital	274 (71.5%)
Community Pharmacy – Large Chain Multiple	50 (13.1%)
Community Pharmacy – Medium/Small Independent Multiple	16 (4.2%)
Community Pharmacy – Independent	14 (3.7%)
Cross-sector programme (both NHS and Community Pharmacy)	29 (7.6%)

### Applicant views and experiences of the application system

When asked about their overall satisfaction with their experiences of shortlisting prospective training programmes, approximately half (*n* = 145, 49.2%) of the survey respondents were satisfied or highly satisfied, with about one in five expressing neither satisfaction nor dissatisfaction (*n* = 56, 19.0%). Higher satisfaction was statistically significantly associated with respondents’ choice of ‘community pharmacy – large chain multiple’ as the highest ranked preference, having received an offer through HEE and the hierarchy of the ranked choices for which offer was received (Table 3). Respondent age, gender, ethnicity and number of training programmes shortlisted were not significantly associated with the satisfaction scores.

**Table 3:** Global satisfaction with the national pre-registration training application process in relation to selection of prospective employers?

Questions	Response options	Overall, how satisfied were you with the preferencing process?*						*P* values^1^
		Very Dissatisfied	Dissatisfied	Neither satisfied nor dissatisfied	Satisfied	Very Satisfied	Total	
Did ‘Community Pharmacy – Medium/Small Independent Multiple’ feature in your programme preferences?	No	21 (11%)	31 (16%)	30 (16%)	93 (49%)	15 (8%)	190 (100%)	*p* = 0.002
	Yes	18 (17%)	24 (23%)	26 (25%)	32 (30%)	5 (5%)	105 (100%)	
	Total	39 (13%)	55 (19%)	56 (19%)	125 (42%)	20 (7%)	295 (100%)	
Did ‘Community Pharmacy – Large Chain Multiple’ feature in your highest ranked programme preferences i.e. your ‘top ten’?	No	26 (11%)	44 (18%)	48 (20%)	110 (45%)	17 (7%)	245 (100%)	*p* = 0.013
	Yes	13 (26%)	11 (22%)	8 (16%)	15 (30%)	3 (6%)	50 (100%)	
	Total	39 (13%)	55 (19%)	56 (19%)	125 (42%)	20 (7%)	295 (100%)	
Did you receive a training place offer through Oriel?	No	10 (32%)	9 (29%)	3 (10%)	8 (26%)	1 (3%)	31 (100%)	*p* = 0.003
	Yes - through clearing	3 (19%)	3 (19%)	4 (25%)	6 (38%)	0 (0%)	16 (100%)	
	Yes in the first round	26 (10%)	43 (17%)	49 (20%)	111 (45%)	19 (8%)	248 (100%)	
	Total	39 (13%)	55 (19%)	56 (19%)	125 (42%)	20 (7%)	295 (100%)	
Which of your preferenced training places were you offered?	20th + ranked choice	12 (18%)	15 (23	17 (26%)	20 (31%)	1(2%)	65 (100%)	*p* = 0.001
	10th to 20th ranked choice	3 (12%)	5 (19%)	3 (12%)	15 (58%)	0 (0%)	26 (100%)	
	4th to 10th ranked choice	4 (11%)	7 (19%)	7 (19%)	14 (38%)	5 (14%)	37 (100%)	
	1st to 3rd ranked choice	7 (6%)	16 (13%)	22 (18%)	62 (52%)	13 (11%)	120 (100%)	
	Total	26 (10%)	43 (17%)	49 (20%)	111 (45%)	19 (8%)	248 (100%)	

*Selection of prospective employers was referred to as ‘preferencing’ in the survey questionnaire. ^1^chi-square test for trend

### Offer outcomes

A total of 264 (89.5%) respondents indicated that they received a training place offer from the national recruitment scheme including 248 (84.1%) in the first round of offer. Of these 233 (93.9%) respondents accepted an offer.

Most respondents (*n* = 181, 72.9%) were either satisfied or highly satisfied with the offer of the training programmes they received. Fifteen respondents (6%) reported declining or letting their offers expire, mostly because of having received an offer outside the national recruitment scheme (Table 4).

**Table 4:** Reasons for declining an offer

Reason for declining an offer	n (%)
Not satisfied with the training place offer	3 (21.4%)
Received another training place offer, outside Oriel	5 (35.7%)
Decided to pursue alternative pre-registration training places outside the national recruitment scheme	4 (28.6%)
Received negative information or feedback about the training place	0
Change in personal circumstances	0
Other	2 (14.3%)

Respondents’ receipt of the offer of a training programme was not significantly associated with the number of training programmes they applied to, respondent age or ethnicity. However, gender was associated with receipt of an offer with female respondents (*n* = 199, 88%) more likely to have received an offer in the first round than the male applicants (*n* = 44, 76%) (chi-square test: *p* = 0.048). Approximately half (*n* = 120, 48.4%) of the respondents reported having received 1st to 3rd ranked preferences. The hierarchy of the received offer was not significantly associated with gender, ethnicity, age or the number of programmes applied by an applicant.

### Quality of information about application process

Most participants indicated high satisfaction with the quality of information about the application process received through their universities or through the applicant handbook (65.8%) (Table 5). Majority (*n* = 217, 73.6%) were satisfied with the length of the time they had available to shortlist their preferred training providers and programmes, however the rest described the timeframe as being inadequate (*n* = 63, 18.1%) or unsure (*n* = 16, 5.4%).

**Table 5:** Participant reflections on their selection of prospective training programmes

Statements	Strongly disagree	Disagree	Neither agree or disagree	Agree	Strongly agree	N/A
Looking back, I feel that I made enough selections in the preferencing process	32 (10.9%)	32 (10.9%)	22 (7.5%)	109 (37.1%)	99 (33.7%)	
I was confident I would receive a training place offer from my final preferences	38 (12.9%)	66 (22.4%)	58 (19.7%)	85 (28.8%)	48 (16.3%)	
Looking back, I feel satisfied with my overall approach to preferencing	40 (13.6%)	46 (15.6%)	44 (14.9%)	103 (34.9%)	62 (21%)	
Based on how I feel I performed in the selection process, my overall performance ranking was expected	51 (17.3%)	73 (24.7%)	65 (22%)	82 (27.8%)	20 (6.8%)	4 (1.4%)
Based on how I feel I performed in the selection process, my training place offer outcome was expected	54 (18.3%)	73 (24.7%)	63 (21.4%)	77 (26.1%)	20 (6.8%)	8 (2.7%)
I believe if I had preferenced differently, I would have been more satisfied with my offer outcome	73 (24.7%)	85 (28.8%)	51 (17.3%)	43 (14.6%)	25 (8.5%)	18 (6.1%)
If I went through the preferencing process again, I would preference differently	64 (21.7%)	74 (25.1%)	35 (11.9%)	67 (22.7%)	47 (15.9%)	8 (2.7%)

### Insights from qualitative data from the questionnaire and focus group

There were eight themes identified from the analysis of qualitative data from both phases of the research, namely: perceived fairness of the national recruitment methodology, views on the assessment components, inclusion of clinical skills assessment, inclusion of communications skills in the selection methodology, listing of training providers in the application system, selecting a training programme and provider during the application process, views on the offer outcomes, accepting or declining an offer. These are reported under three categories below:

### Applicant views on the selection methodology

#### Perceived fairness of the national recruitment methodology

Qualitative data provided insight into the student views on the national recruitment process. Students who often expressed satisfaction with the offer outcome perceived the system as being fair and offering equal opportunity to obtain a training place for all candidates.

‘The system has massive advantages: it gives opportunity to everyone equally and reduces the number of friendship based recruitment that was happening in many hospitals as well as community pharmacy.’ [Demography data not available].

Students who were generally less satisfied with the recruitment process mainly referred to the lack of opportunity to locally negotiate training programmes. Some also referred to the perceived lack of inclusion of academic performance and placement experiences in the assessment process.

#### Views on the assessment components

The selection examination, consisting of the SJT, MMI and pharmaceutical calculations, was commended for its ability to distinguish candidate abilities. Most participants however, suggested that previous work experience, academic achievement and extra-curricular activities should be given weighting during the selection process.

‘I feel that you should take into consideration applicants’ CVs and previous experience in the future. Because although they are assessed by the answers you give on the day, one mistake in the very short interview process due to nerves could cost you a pre-registration position. When in reality you have more than enough experience and skill to perform in that role.’ [Female, 22].

‘I feel students that have done well academically should have some sort of advantage over others in the application process.’ [Male, 22].

#### Inclusion of clinical skills assessment

Some participants suggested that the selection process should incorporate objective assessment of clinical knowledge and skills.

‘Incorporate clinical knowledge assessment into the selection process. However the current selection criteria focus solely on different aspects of situational judgement, which means the applicants accepted within acute settings may not necessarily possess satisfactory clinical knowledge.’ [Male, 25].

‘Application of clinical knowledge to patient-based scenarios should be incorporated to allow students to demonstrate the skills required to be a good pharmacist.’ [Male, 22].

#### Inclusion of communications skills in the selection methodology

There were suggestions that communication skills should have further bearing on the assessment process as some perceived that assessment focussed more on the ‘content’ aspect of their consultation.

‘Need more assessment on communication skills as only the content of what was said in the MMIs was marked, not how they conducted themselves.’ [Female, 21].

Some expressed the view that separate outcomes should be assessed for those opting for hospital only or community pharmacy only pre-registration training programmes. Participants suggested that as the SJT and numeracy tests needed no interactions with the interviewers, they could be held remotely at regional hubs/test centres.

### Applicant views and experiences of the application process

#### Listing of training providers in the application system

Most commended the way by which training providers were listed on the application system. However there were suggestions that geographical information about the training providers should be made clearer. Exact locations of the training providers were often difficult for applicants to decipher.

‘A map function may be useful. If you are applying for *** (a large pharmacy chain) in Greater Manchester/Lancashire, this covers quite a few branches. And had the exact location been available my preferences would likely have been very different, as I would be able to be more specific.’ [Male, 21].

#### Selecting a training programme and provider during the application process

Participants were generally positive about their experiences of the application tool to facilitate the selection of their preferred programmes. Most demonstrated an understanding of how the application system worked and spoke highly of how the listing of the employers and filtering system were laid out.

‘I thought it was really well done in the sense of, it had literally every single place on it and the timeframe you were given allows you to like literally consider all the places’. [Male, 23].

‘I think the filtering system was actually really good and useful cause otherwise, if you’re just scrolling and scrolling through all these different places, it gets quite confusing and you can easily miss something that you may have wanted to preference.’ [Male, 23].

Participants described how they would approach shortlisting their preferred training programmes if they had the opportunity to do so again. Some mentioned that they would rank more of the hospital pre-registration training programmes in their top choices given the more competitive nature of the hospital pre-registration training programmes.

Many participants suggested that more time was needed during the application window to shortlist their preferred training programmes.

‘Once you have preferenced, I think there should be the option to edit them after saving. This should have no need to be fully submitted months before the assessment process’ [Female, 23].

Some also suggested that it would be better if applicants were able to see the popularity of each training site given the number of students who have selected them in order to inform their decision making.

### Applicant views and decisions on the offer outcomes

#### Views on the offer outcomes

Participants expressed a range of emotions in describing their perceptions of the offer outcomes. These included outcomes ‘better than expected’, ‘as expected’ or ‘worse than expected’.

‘The way they had said, like, preference so many; I was surprised to get something so high but, I was happy but I was, it was a surprise as well’ [Female, 22].

Some participants mentioned that the outcomes in the selection centre exams did not match their performance at their university.

‘Students who did nothing throughout the 4 years and got great placements, while students who spent their whole summer gaining experience received no offer.’ [Demography data not available].

#### Accepting or declining an offer

Participants described weighing up their offer in the context of perceived quality of training programme and career prospects after the training.

‘I’m quite excited by the fact it’s going to be something new and, I know it’s a place where I will be, like, pushed to work hard and achieve more than I may have if I’d chosen somewhere, say at a community pharmacy where I was just, I knew pretty much what I was going to get and I’m also, it’s also the fact that I know it’s quite a reputable place and then I think it’s the job prospects after that’ [Male, 23].

Some participants mentioned the use of upgrade functions in the application system. However, some were apprehensive that opting for upgrades would mean losing ‘control’ of their current offers.

‘For me to go into upgrade it was like letting go of control of the position I was given and I didn’t want to give up that control, which is why I didn’t go into upgrade’ [Female, 22].

## Discussion

This is the first large scale evaluation of pharmacy students’ early experiences with the national pre-registration pharmacist training recruitment scheme in England and Wales. Study participants were generally satisfied with the national recruitment scheme. In regards to perceived fairness and components of selection methodology, there were suggestions from the participants to consider academic merit and placement experiences to have direct bearing on the selection process. This is in contrast to the findings from the research with medical students where they often prefer interviews based selection to cognitive aptitude tests [9, 13, 14]. Recruitment of foundation trainee doctors in the UK uses educational performance measures (EPM) in their selection and ranking of candidates [15]. The EPM is a measure of clinical and non-clinical skills, knowledge and educational performance up to the point of application to the Foundation Programme. This considers medical school performance including clinical and non-clinical performances; and educational achievements including additional degrees and publications. Longitudinal evaluation of candidate views on the selection methodology in UK general practice trainees suggested that assessment methods using simulated patient scenarios were considered most relevant to candidates clinical training and future roles by the applicants [16].

Participants alluded to various factors linked to their satisfaction or dissatisfaction to the recruitment process. Reasons for satisfaction included convenience of the opportunity to submit applications to multiple employers and the ability of the selection methodology to distinguish candidate abilities. Dissatisfaction was linked to lack of opportunity to locally negotiate training programmes. Qualitative data suggests that, given the number of training programmes available, there was a need to extend the decision making timelines and improve the technical tool to support geographical information of prospective programmes. These changes are likely to positively influence greater satisfaction with the national recruitment process.

Participants of this study described selecting high number of prospective training programmes from the long list available to them. Published literature shows that perception about how far a training programme meets applicants’ personal career goals is an important factor in how students select prospective training programmes [17, 18]. Therefore, having a career goal at the stage of making a pre-registration training application is important from student perspectives [19]. Particularly, the lack of an adequate number of training programmes that allow pharmacy pre-registration trainees to work in multiple sectors may mean that those students without a career goal at that stage may often find the application process more challenging [19]. Stability of decisions [20] is an area warranting further study as little is known about how far pharmacy graduates stay in the sector chosen for their pre-registration training programme as their career progresses.

### Strengths and limitations

This is the first large scale evaluation of early experiences of pharmacy students on the national pharmacist pre-registration training programme in the UK. The responses to the survey and focus groups were, however, low. While we assumed data saturation in relation to the qualitative data from survey and focus groups, the low response rate of the survey limits the external validity of the findings. We compared the survey respondents with the demography of the national applicant data which suggested that respondents were comparable with regards to the sex distribution (total females amongst 2694 national applicants were 64.8% vs 76.0% in our survey). The low response can be explained by data collection being conducted during the Master of Pharmacy final year exam period for most of the Universities. Due to the time-sensitive nature of the evaluation to inform the subsequent cycle of recruitment, the researchers could not wait for a later opportunity. Also, there may have been differences in the level of engagement with the invitation between schools of pharmacy as the response rate varied across schools. The survey was conducted after the offer outcomes were released, which could have influenced satisfaction and dissatisfaction rates. Those who obtained an offer from their higher ranked preferences were more likely to express higher satisfaction.

### Implications for practice

Experiences of pharmacy students of the national recruitment scheme suggests that students regard the scheme as fair with a high majority of the respondents satisfied with their offer outcomes. Widening the timeframe allowed for employer shortlisting by the applicants, improved methods of employer listing in the application system, and greater transparency in the geographical location of the training places can improve applicant satisfaction with the application process.

It will also be useful to repeat the evaluation in the future to address ongoing needs. Long-term evaluations will enable consideration of how career aspirations of pharmacy students change over time given the greater clinical roles and diversification of pharmacy workforce in relation to recent policy initiatives. There is also a need to undertake an evaluation of the national recruitment scheme from the perspective of wider stakeholders.

There is a need to gather evidence on the long term predictive validity of the selection methodology. This could include investigating links between student performances in the selection examination, their training performance and their outcome in the General Pharmaceutical Council (GPhC) registration assessment. A recent evaluation of long term predictive validity showed that EPM and SJT scores provide a good predictive validity for completion of training programme by the candidates [20]. There is a need to undertake a long term predictive validity study of the new selection methodology applicable to pharmacy. In particular, candidate performance in their pre-registration training, General Pharmaceutical Council pre-registration examination and tutor rating of trainee performances could be appropriately linked to candidate performance in the national training selection examination.

Participants of this study were generally satisfied with the information they had received from the University around the national recruitment process. From the Universities’ perspectives, while the recruitment and associated assessment are externally-led, the national recruitment process is expected to have implications on the initial education and academic training of pharmacy students. The academic curriculum need to align with the professional attributes framework and person specification being used in the national recruitment assessment methodology to maximise student performances. It is imperative that University perspectives are investigated in future evaluations.

## Conclusion

Experiences of pharmacy students of the national recruitment scheme suggests that most students regard the scheme as fair with a high majority of the respondents satisfied with their offer outcomes. Respondents in this study positively regarded the application process of the national recruitment system for the convenience it allows students to apply to multiple pre-registration training providers. Low response rate limits generalisability of the findings. Changes in the selection assessment methodology will require consideration of evidence based approaches.

## Supplementary information

**Additional file 1.** Consolidated criteria for reporting qualitative studies (COREQ): 32-item checklist.

## Data Availability

The datasets used and/or analysed during the current study are available from the corresponding author on reasonable request.

## Abbreviations

EPM: Educational Performance Measures
GPhC: General Pharmaceutical Council
HEE: Health Education England’s
MMI: Multiple Mini Interviews
OSPAP: Overseas Pharmacist Assessment Programme
SJT: Situational Judgement Tests
